# Quality assessment and data handling methods for Affymetrix Gene 1.0 ST arrays with variable RNA integrity

**DOI:** 10.1186/1471-2164-14-14

**Published:** 2013-01-16

**Authors:** Katie S Viljoen, Jonathan M Blackburn

**Affiliations:** 1 Institute of Infectious Disease and Molecular Medicine, University of Cape Town, Anzio Road, Observatory, Cape Town, 7925, South Africa

**Keywords:** Gene expression profiling, Microarray, RNA quality, RNA integrity number, Quality control, ComBat, Surrogate variable analysis, Non-biological experimental variance

## Abstract

**Background:**

RNA and microarray quality assessment form an integral part of gene expression analysis and, although methods such as the RNA integrity number (RIN) algorithm reliably asses RNA integrity, the relevance of RNA integrity in gene expression analysis as well as analysis methods to accommodate the possible effects of degradation requires further investigation. We investigated the relationship between RNA integrity and array quality on the commonly used Affymetrix Gene 1.0 ST array platform using reliable within-array and between-array quality assessment measures. The possibility of a transcript specific bias in the apparent effect of RNA degradation on the measured gene expression signal was evaluated after either excluding quality-flagged arrays or compensation for RNA degradation at different steps in the analysis.

**Results:**

Using probe-level and inter-array quality metrics to assess 34 Gene 1.0 ST array datasets derived from historical, paired tumour and normal primary colorectal cancer samples, 7 arrays (20.6%), with a mean sample RIN of 3.2 (SD = 0.42), were flagged during array quality assessment while 10 arrays from samples with RINs < 7 passed quality assessment, including one sample with a RIN < 3. We detected a transcript length bias in RNA degradation in only 5.8% of annotated transcript clusters (p-value 0.05, FC ≥ |2|), with longer and shorter than average transcripts under- and overrepresented in quality-flagged samples respectively. Applying compensatory measures for RNA degradation performed at least as well as excluding quality-flagged arrays, as judged by hierarchical clustering, gene expression analysis and Ingenuity Pathway Analysis; importantly, use of these compensatory measures had the significant benefit of enabling lower quality array data from irreplaceable clinical samples to be retained in downstream analyses.

**Conclusions:**

Here, we demonstrate an effective array-quality assessment strategy, which will allow the user to recognize lower quality arrays that can be included in the analysis once appropriate measures are applied to account for known or unknown sources of variation, such as array quality- and batch- effects, by implementing ComBat or Surrogate Variable Analysis. This approach of quality control and analysis will be especially useful for clinical samples with variable and low RNA qualities, with RIN scores ≥ 2.

## Background

RNA degradation is a common concern in gene expression analysis, especially for clinical samples where RNA degradation may occur before sample collection [1]. A wealth of archival material, either snap frozen or formalin fixed and paraffin embedded (FFPE), could potentially be used for gene expression analysis, given an appropriate method to evaluate and account for the effect of RNA degradation on the quality of downstream gene expression data. Methods such as the RNA integrity number (RIN) algorithm reliably assesses RNA integrity by extracting features from the RNA electropherogram. The RIN algorithm was developed using learning tools to identify regions (features) indicative of RNA integrity in the electropherogram, which are then used to compile the RNA integrity number on a scale of 1 to 10. However, the relevance of RNA integrity in gene expression analysis, especially when there is large variability between samples, requires further investigation and validation on a platform specific basis. The impact of RNA integrity on gene expression analysis has been investigated on both qRT-PCR and certain microarray platforms [2-7]. Opitz et al investigated the impact of RNA degradation on Agilent 44 k gene expression profiling by subjecting RNA from clinical biopsies to temperature-induced RNA degradation and comparing gene expression to the original, intact samples. Notably, less than 1% of genes were affected, even after substantial RNA degradation, where control and test samples had RINs of 9 and 5 respectively. The affected transcripts were relatively shorter, had lower GC content, or had probes relatively closer to the 5' region of the gene compared to more robust genes [6]. Although the process of RNA degradation is not fully understood, both exonuclease and endonuclease activity is likely to play an important role [6]. Classical oligo-dT based cDNA synthesis, which starts at the poly-A tail, will most certainly be compromised by exonuclease activity. In contrast random priming does not rely on full length mRNA and therefore is in theory at least partially relieved from the affects of RNA degradation [6-9].

When using semi-degraded RNA for gene expression studies, reliable measures of array quality provide valuable information that can be used to guide downstream analysis. Microarray data quality may be defined in terms of accuracy (systematic bias between the true and measured value), precision (the uncertainty in replicated measures), specificity (the selective power of the measurement to respond only to the specific targets) and sensitivity (the expression range potentially covered by the measurement) [10]. Any attempt to utilise array quality results to guide downstream analysis should ideally take into account the possible effects of RNA degradation on sensitivity, specificity and accuracy. In previous work, Binder et al proposed a single-array preprocessing method that allows correction for systematic biases such as RNA degradation by utilising information on the 3'/5'-amplification bias and the sample-specific calling rate [10]. Lassmann et al proposed using a data adjustment method to allow comparative analysis of microarray datasets derived from fresh frozen vs. FFPE samples by centering the log intensities of each probe set independently to a mean of zero in both groups [8]. Chow et al evaluated the suitability of different quality control and preprocessing strategies for use with partially degraded RNA samples on the Illumina DASL-based gene expression assay using mean inter-array correlation and multivariate distance matrix regression (MDMR) as a measure of success [11]. Unfortunately none of these studies are directly applicable to one of the most commonly used human transcriptomic microarray platforms, namely Affymetrix Gene 1.0 ST arrays, either because they do not use a random priming approach or because the design of the microarray platform differs substantially from Gene 1.0 ST arrays. We therefore identified two alternative approaches that might be used as compensatory methods: Firstly, Johnson et al developed an empirical Bayes algorithm, ComBat, to directly adjust for non-biological experimental variation. As the name implies, this method is most often used to adjust for batch effects i.e. when microarrays are processed on different dates [12]. Secondly, Leek et al developed a method called Surrogate Variable Analysis (SVA), which examines the contribution of sources ofsignal due to unknown (surrogate) variables in high-dimensional data sets, which may confound the biological signal of interest [13]. The surrogate variables are constructed directly from the gene expression data where groups of genes that are affected by each source of variation are identified, factors are then estimated for each array which can be included in a linear model to adjust for unknown sources of noise e.g. RNA- or array-quality.

Here, we investigate the relationship between RNA integrity and array quality on Affymetrix Gene 1.0 ST arrays for 34 paired colorectal tumour and adjacent normal biopsies of highly variable RNA integrity. We assume that at a certain point on the RIN scale, RNA will be degraded to the extent where fragments are too small to analyse reliably and for the purpose of this analysis we arbitrarily select a RIN cutoff of 2. We describe the within- and between-array quality control measures and analysis methods that we found most relevant for gene expression analysis of samples with highly variable RINs on Affymetrix Gene 1.0 ST arrays. We then investigate the possibility of a transcript-length dependency in RNA degradation. Finally, we apply array quality information to either exclude quality-flagged arrays, to directly adjust the data using the ComBat algorithm, or to account for unknown sources of variation (such as RNA integrity or array quality) in the model fitting process using SVA. The data discussed, have been submitted to ArrayExpress, with accession number E-MEXP-3715.

## Results

### Array quality

**Figure 1:**
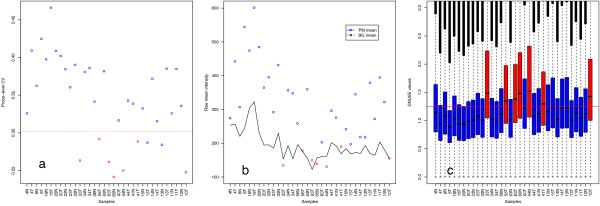
**Array quality metrics. a**) Raw coefficient of variation across all probes by sample, the red line represents our chosen threshold which is calculated as 2SD from the mean of CVs for arrays with RINs > 6. **b**) Raw perfect match mean - background mean **c**) Global normalised unscaled errors (GNUSE) across probes for each array. Samples that were flagged during quality assessment are highlighted in red.

We assessed array quality using within- and between-array measures – the former to assess raw data quality (Figure 1a &1b), and the latter to assess the quality of an array relative to a large publically available collection of high quality Gene 1.0 ST arrays (Figure 1c). Raw array quality was investigated at the probe level by calculating the difference between the means of perfect match- and background-probes for each array as well as the coefficient of variation (CV) across all probes for each array. Preprocessed data quality was assessed using the global normalised, unscaled standard error (GNUSE) [14]. See Methods section for details.

The 34 RNA samples used in this study had a mean RIN of 6.3 and a standard deviation of 2.0. Samples that failed all three measures of quality had RINs between 2 and 3.3 as summarised in Table 1. Samples were ranked by GNUSE median and we found a good concordance in terms of ranking between the different quality control metrics. Samples that failed at least two out of the three quality measures were flagged for downstream analysis, resulting in 7 out of 34 samples being flagged (mean RIN = 3.2; SD = 0.42). Interestingly, for one sample with a RIN of 2.6, array quality was not compromised, judged by our quality measures. The possibility of a RIN-independent RNA quality factor, such as chemical purity, was investigated by performing a two-tailed Student’s t-test, comparing A260/230 ratios between quality-flagged and quality-passed sample groups but no significant association was found (p-value = 0.14).

**Table 1:** Array quality assessment summary

**Sample ID**	**RIN**	**RNA 260/230 ratio**	**GNUSE**	**probe-level CV**	**PM-BG**	**Array weight**
**44N**	3	2.41	**fail (1)**	**fail (3)**	**fail (1)**	**0.22 (1)**
**33T**	2.8	2.08	**fail (2)**	**fail (5)**	**fail (2)**	**0.28 (2)**
**60N**	3.2	2.03	**fail (3)**	**fail (1)**	**fail (3)**	**0.42 (3)**
**63T**	3	2.2	**fail (4)**	**fail (4)**	**pass**	**0.59 (6)**
**10T**	3.2	2.18	**fail (5)**	**fail (2)**	**fail (4)**	**0.60 (7)**
**56T**	3.3	1.87	**fail (6)**	**fail (10)**	**fail (5)**	**0.42 (4)**
**41T**	4.2	2.21	**fail (7)**	**fail (9)**	**pass**	**0.78 (8)**
13N	4.6	2.24	pass	fail (7)	pass	0.82 (9)
15T	4.8	2.15	pass	fail (8)	pass	1.07 (15)
4N	2.6	1.62	pass	pass	pass	0.44 (5)
18N	7.1	1.66	pass	pass	pass	0.83 (10)
8T	8.5	2.16	pass	pass	pass	0.85 (11)
56N	6.5	1.94	pass	pass	pass	0.95 (12)
20T	7.4	1.6	pass	pass	pass	1.02 (13)
44T	6.9	1.72	pass	pass	pass	1.03 (14)
11T	8.6	2.16	pass	pass	pass	1.07 (16)
60T	6.4	1.64	pass	pass	pass	1.09 (17)
14T	6.4	1.76	pass	pass	pass	1.09 (18)
13T	8.3	2	pass	pass	pass	1.11 (19)
23T	7	2.17	pass	pass	pass	1.18 (20)
8N	7.1	2.22	pass	pass	pass	1.25 (21)
18T	7.4	1.85	pass	pass	pass	1.26 (22)
33N	8.1	1.82	pass	pass	pass	1.45 (23)
34T	8	2.25	pass	pass	pass	1.49 (24)
11N	6.8	1.94	pass	pass	pass	1.50 (25)
20N	7.3	2.11	pass	pass	pass	1.50 (26)
63N	7.5	2.13	pass	pass	pass	1.61 (27)
23N	8.4	2.02	pass	pass	pass	1.61 (28)
34N	8.3	2.21	pass	pass	pass	1.61 (29)
14N	8.1	2.36	pass	pass	pass	1.74 (30)
41N	5.4	2.07	pass	pass	pass	1.76 (31)
10N	7.3	1.78	pass	pass	pass	1.78 (32)
15N	6.9	2.16	pass	pass	pass	1.90 (33)
4T	8.4	2.25	pass	pass	pass	2.14 (34

Array performance is ranked for each measure with 1 considered the worst quality. Samples highlighted in bold were flagged for downstream analysis.

### Transcript-dependent effects of RNA degradation on accuracy

**Figure 2:**
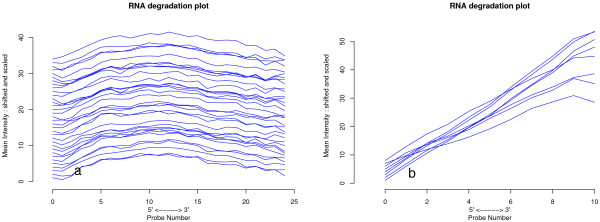
**Mean probe intensity by probe position. **Each line represents an array for **a**) Gene 1.0 ST arrays: transcript clusters with exactly 25 probes (N = 4644) and **b**) HGU133-plus2 arrays previously analysed with a subset of the cohort: probesets with exactly 11 probes per probeset.

To investigate a possible probe-positional intensity bias related to RNA integrity, we plotted the mean probe intensity from the 5'- to 3' end of the sequence using 4644/32321 (14.4%) of transcript clusters for Gene 1.0 ST arrays and 54130/54675 (99%) of probesets for HGU133-plus2 arrays. The number of probes per set varies for GeneST arrays, so we selected the largest group (N = 4664), which had exactly 25 probes/set. Interestingly, from the 4644 transcript clusters displayed in Figure 2, Gene ST 1.0 arrays, do not display the same probe-positional intensity bias typically seen in oligo-dT based arrays such as the HGU133-plus2 arrays.

**Figure 3:**
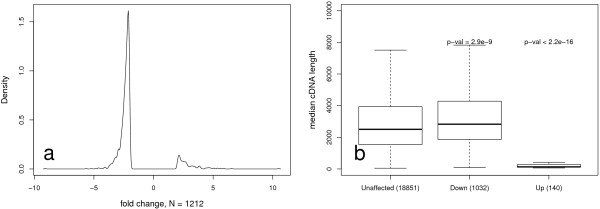
**Characteristics of genes most affected by RNA degradation. **Comparison of samples that either passed or were flagged during QC. **a**) Fold change distribution of annotated transcript clusters comparing samples that were flagged vs. samples that passed QC **b**) Gene lengths of uniquely identified genes. Expression signal significantly increased (Up) or decreased (Down) with respect to the ‘Unaffected’ group, judged using a Mann-Whitney test. Adjusted p-value ≤ 0.01, |fold change| > 2.

We next investigated which genes were most affected in our quality-flagged category and identified 1994 out of 21943 annotated transcript clusters (with 1172 uniquely identified genes) that were significantly different (fold change ≥ |2|, adjusted p-value ≤ 0.05) between the two quality categories previously discussed. Of the 1172 uniquely identified genes, 1032 and 140 showed decreased or increased intensity in the quality-flagged category respectively (Figure 3a). To investigate transcript characteristics in the genes most affected, we compared transcript lengths (taken as the median cDNA length for each gene) between the different groups. Compared to the unaffected genes, median cDNA lengths of genes that showed increased intensity were significantly shorter (p-value < 2.2 *e* − 16) while those with decreased intensity significantly longer (p-value = 2.9 *e* − 9) with regards to quality, judged using the Mann Whitney test (Figure 3b).

### Quality dependent methods of data adjustment and analysis

After assigning samples to two categories according to array quality measures, we next assessed the performance of the five preprocessing and analysis methods. Broadly speaking, the data was either directly adjusted for quality effects using ComBat, or quality-flagged samples were excluded from the analysis, or possible quality effects were addressed by including known or unknown sources of non-biological variance in the linear model fit to assess differential expression.

The five methods of data preprocessing and analysis, further detailed in the Methods section, were: 1) Estimating array quality weights which were then included in the linear model fit; 2) Excluding quality-flagged arrays from the analysis; 3) Applying a batch correction algorithm, ComBat, [12] to directly adjust the data according to quality, where arrays were divided into two categories according to the array quality assessment; 4) “Quality” and “batch” were included as a factors in the linear model together with disease status; 5) Possible unknown sources of non-biological variance, such as quality, was estimated by SVA, with the output incorporated into the linear model fit [13].

**Figure 4:**
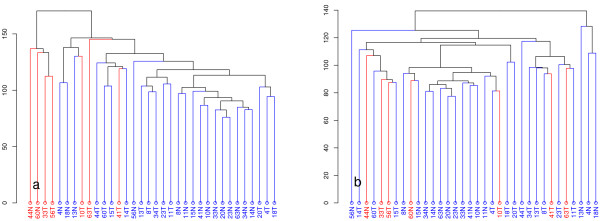
**Expression profiles of samples clustered using average linkage hierarchical clustering. a**) Sample clustering after preprocessing. **b**) Sample clustering after preprocessing and correction for batch and quality using ComBat. Samples that were flagged during quality assessment are highlighted in red. The dissimilarity measure (height) used was 1- Pearson correlation of the log_2_-transformed expression values.

**Figure 5:**
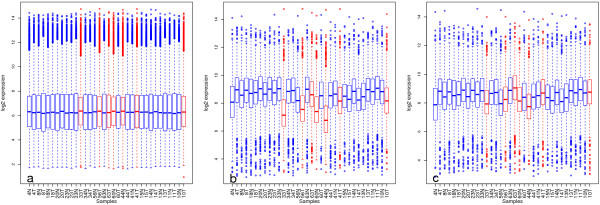
**Boxplots of frma expression. a**) All transcript clusters. **b**) Genes most affected by quality (adjusted p-value ≤ 0.01, |fold change| > 2. **c**) Samples that were flagged during quality assessment are highlighted in red.

To assess the effect of using ComBat for direct data adjustment, hierarchical clustering using Euclidian distance was performed before and after direct adjustment (Figure 4). We chose to use Euclidian distance based on research by Gibbons et al who demonstrated that, for log-transformed expression data, using Euclidian distance is more appropriate than Pearson’s correlation coefficients [15]. Before adjustment, samples that were flagged during quality assessment cluster closely together, irrespective of the disease status of the samples. After adjustment, the maximum distance between samples is greatly reduced, and quality-flagged samples no longer cluster together. Also, samples segregate more clearly by disease status after adjustment. Furthermore, applying ComBat clearly has a stabilising effect on the transcript clusters most affected by RNA quality (Figure 5b &5c).

**Figure 6:**
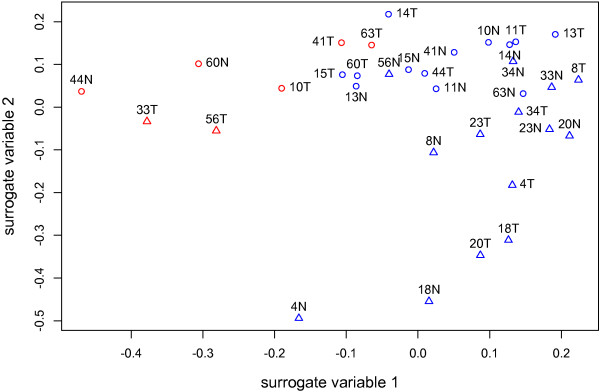
**Surrogate variable analysis results. **Samples that were flagged during quality assessment are highlighted in red. Two latent variables were identified by SVA. Circles and triangles represent samples from two different batches.

SVA identified two surrogate variables that were subsequently used in downstream analysis. Plotting the estimates of these surrogate variables for each sample revealed a pattern whereby samples were clearly grouped by batch and quality (Figure 6). Importantly, SVA identified these two variables without supervision.

To evaluate the performance of each method, we first compared the number of differentially expressed genes detected between tumour and normal samples at a stringent p-value of 0.01. For our analysis, we did not use a fold change cutoff since we feel that artificial fold change cutoffs, which exclude subtle changes in the expression of many genes, may result in the loss of valuable biological information, or worse, affect the interpretation of the data – this is particularly true for applications such as network/pathway analysis [16].

**Figure 7:**
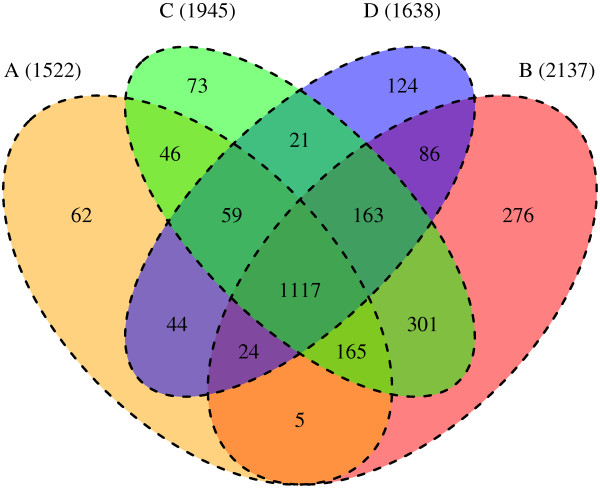
**Venn diagram of unique differentially expressed genes (tumour vs. normal) with adjusted p-values ≤ 0.01 for the four best-performing methods. A** - removing quality-flagged arrays before analysis. **B** - applying SVA to batch corrected data. **C** - ComBat used to correct for batch and quality. **D** - Array weights included in the linear model.

SVA and ComBat detected 2137 and 1945 genes (p-value ≤ 0.01), respectively. The top four methods had 1117 differentially expressed genes in common (Figure 7). At the commonly used p-value- and fold change-cutoffs of 0.05 and 2 respectively, SVA, Combat, ArrayWeights and excluding arrays, produced 447, 475, 461 and 521 differentially expressed genes respectively, suggesting similar performance under these criteria. We next assessed the relevance of these differentially expressed genes in colorectal cancer using Ingenuity Pathway Analysis where, statistically significant over-representation of our listed genes in a given process such as “colorectal tumour” or “infection of embryonic cell lines” is scored by p-value.

We considered the top 10 functions for each method (Table 2) from which it was clear that the 615 and 423 additional genes identified as differentially expressed by SVA and ComBat, compared to that obtained when excluding quality-flagged arrays, were certainly relevant to colorectal cancer. Using IPA, we considered the top 10 upstream regulators (highest absolute activation z-scores) when comparing tumour vs. normal samples, to further investigate the utility of SVA or ComBat as suitable analysis methods when including low-RIN samples (Table 3). We found considerable overlap in the identity and direction of activation of these upstream regulators between the methods compared.

**Table 2:** P-values for evidence for overrepresentation in the functions listed for each method

**Functions**	**A**	**B**	**C**	**D**	**E**
Cancer	7.72E-29	NA	8.15E-24	NA	3.38E-23
cancer	NA	2.58E-25	NA	2.70E-26	NA
carcinoma	8.64E-37	2.52E-33	1.87E-34	2.87E-32	5.56E-30
colon cancer	1.30E-26	1.19E-36	1.10E-26	1.99E-21	3.29E-21
colon tumor	1.10E-26	4.31E-37	3.65E-27	1.80E-21	7.28E-22
colorectal cancer	2.27E-26	4.74E-29	2.43E-26	1.11E-21	1.98E-23
colorectal tumor	2.28E-26	6.80E-29	2.97E-26	4.67E-22	1.72E-23
digestive organ tumor	2.68E-31	6.82E-32	1.24E-28	7.27E-27	2.72E-29
epithelial tumor	2.16E-38	NA	2.27E-35	NA	1.11E-30
gastrointestinal tract cancer	2.35E-25	2.42E-28	4.00E-24	3.19E-21	5.31E-22
intestinal cancer	2.02E-26	5.77E-29	2.58E-26	1.03E-21	1.55E-23
neoplasia	NA	1.63E-24	NA	1.10E-25	NA
solid tumor	3.31E-35	8.07E-32	6.80E-33	4.65E-31	3.88E-29
tumorigenesis	NA	1.55E-26	NA	3.31E-28	NA
uterine serous papillary cancer	3.46E-21	1.71E-20	8.61E-25	1.26E-22	1.14E-15

A - excluding quality-flagged arrays from the analysis. B - applying SVA to batch corrected data. C - ComBat used to correct for batch and quality. D - Array weights included in the linear model. E - including batch and quality as factors in the linear model.

**Table 3:** Top 10 IPA-derived upstream regulators, by absolute activation z-score

**A**					
**Upstream Regulator**	**Log Ratio**	**Molecule Type**	**Predicted Activation State**	**Activation z-score**	**p-value of overlap**
TP53		transcription regulator	Inhibited	−4.88	1.05E-16
CDKN1A	−0.469	kinase	Inhibited	−3.274	4.20E-10
TRAF2		enzyme	Activated	2.804	3.06E-06
CCNK		other	Activated	2.905	3.83E-04
TNF		cytokine	Activated	2.935	7.69E-04
IL1B		cytokine	Activated	2.952	1.76E-01
TP63		transcription regulator	Activated	3.181	8.37E-10
TREM1		other	Activated	3.352	3.69E-05
FOXM1	1.37	transcription regulator	Activated	4.28	3.71E-17
Mek		group	Activated	4.336	2.38E-07
**B**					
**Upstream Regulator**	**Log Ratio**	**Molecule Type**	**Predicted Activation State**	**Activation z-score**	**p-value of overlap**
TP53	0.622	transcription regulator	Inhibited	−5.749	6.48E-12
TGM2		enzyme	Inhibited	−4.243	3.64E-02
CDKN1A	−0.485	kinase	Inhibited	−3.548	1.85E-10
KDM5B		transcription regulator	Inhibited	−3.126	3.31E-08
NFkB (complex)		complex	Activated	3.034	3.59E-03
TREM1		other	Activated	3.073	2.18E-05
TP63		transcription regulator	Activated	3.63	6.25E-06
IL1B		cytokine	Activated	3.686	4.13E-01
FOXM1	1.29	transcription regulator	Activated	3.925	5.82E-11
Mek		group	Activated	4.771	7.08E-08
**C**					
**Upstream Regulator**	**Log Ratio**	**Molecule Type**	**Predicted Activation State**	**Activation z-score**	**p-value of overlap**
TP53		transcription regulator	Inhibited	−5.126	1.30E-13
CDKN1A	−0.496	kinase	Inhibited	−3.534	5.99E-10
TGM2		enzyme	Inhibited	−3.402	4.25E-02
miR-483-3p		mature microRNA	Inhibited	−3.153	6.49E-03
EGFR		kinase	Activated	3.104	4.43E-03
IL1B		cytokine	Activated	3.281	1.73E-01
TP63		transcription regulator	Activated	3.524	1.48E-09
TREM1		other	Activated	3.845	5.74E-06
FOXM1	1.398	transcription regulator	Activated	4.386	4.18E-16
Mek		group	Activated	4.654	9.72E-08

A - excluding quality-flagged arrays from the analysis. B - applying SVA to batch corrected data. C - ComBat used to correct for batch and quality.

### qRT-PCR validation of select genes

**Figure 8:**
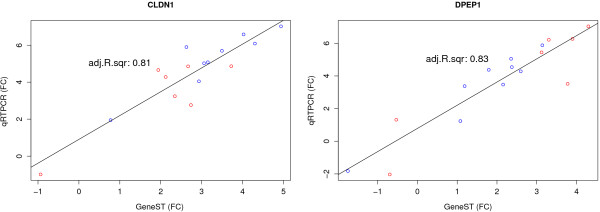
**DPEP1 and CLDN1 tumour vs. normal fold change (FC) results for qRT-PCR and microarray results. **Samples that were flagged during quality assessment are highlighted in red.

In order to ascertain whether or not data obtained by microarray analysis with low-RIN samples were comparable to the results obtained using the method designed by Antonov et al for qPCR analysis of low-RIN samples, we selected two genes, dipeptidase 1 (DPEP1) and claudin 1 (CLDN1), for qRT-PCR validation. Given that our microarray data analysis suggests ~95% of genes are unaffected by RNA integrity, we wished to compare microarray and qPCR data for genes that were apparently unaffected by RNA integrity; DPEP1 and CLDN1 were found to be significantly differentially expressed in our microarray data by all of the five methods used and, in addition, there is strong literature evidence for their differential expression between tumour and normal samples. From reference genes previously cited as suitable for colorectal cancer studies, we selected those most stably expressed in our cohort using the Normfinder algorithm (UBC, B2M, ATP5E) [17-21]. We found good correlations, for both CLDN1 (Adjusted R^2^ = 0.81) and DPEP1 (Adjusted R^2^ = 0.83), between qRT-PCR- and microarray-based fold change values (Figure 8), irrespective of RIN score.

## Discussion

RNA is extremely vulnerable to degradation and as such has the potential to introduce a systematic bias in gene expression measures. Reliable measures of sample and data quality are therefore essential to evaluate the effects of RNA integrity on accuracy, sensitivity and specificity of gene expression results. From previous studies as well as our own, it is now clear that the level of acceptable RNA degradation within an experiment depends largely on the experimental design, platform and application. Multiple studies have demonstrated an improvement in microarray and qRT-PCR performance by using random priming when RNA integrity is in doubt. Here we observed a direct association between RINs and array quality in the majority of cases. To gauge the consequences of using these arrays in downstream analysis, we compared quality-flagged to quality-passed arrays and found a relatively small subset of genes, 1172/20019, to be significantly affected (p-value 0.05, FC ≥ |2|) in our samples on the Gene 1.0 ST platform. It is of course possible that the exact identity and proportion of the affected genes may differ between studies on Gene 1.0 ST arrays but, based on our data, we suggest that the overall proportion of affected genes is unlikely to be significantly different to that observed here. Depending on the application, this may or may not have an effect on the study outcome. However, the most common microarray applications such as finding differentially expressed genes between two conditions, pathway analysis, and clustering do not rely on interrogating specific genes and appear to be largely robust to the effects of RNA degradation on this platform (Table 2).

Using within- and between-array quality measures, we investigated the relationship between RNA integrity and array quality on Affymetrix Gene 1.0 ST arrays. We found a combination of within- and between-array quality measures useful to rank samples by array quality. However, the single most useful array quality measure appears to be GNUSE, since it provides a more general measure of array quality relative to a large set of publically available arrays. We found that 86% of samples with RINs ≤ 3.3 were flagged by at least two of our quality control measures. One sample with RIN score < 3 passed all three quality measures, although it did have relatively low array quality weight. Furthermore, 10 out of 17 samples with RIN scores ≤ 7 passed at least 2 out of 3 quality measures, suggesting that the widely used RIN cutoff of 7 is too stringent for Gene 1.0 ST arrays.

We then examined the genes most affected by RNA degradation and demonstrated a relationship between accuracy and length of the original transcript, with both longer than average, and very short transcripts being under- and overrepresented in quality-flagged samples respectively. This is in contrast to the findings by Opitz et al who found that short transcripts were more vulnerable to the perceived effects of degradation, whereas long transcripts were more stable relative to the average length transcript [6]. Interestingly, of the genes that were overrepresented in quality-flagged samples, 70% were small non-protein coding RNAs, including 94 small nucleolar RNAs, and 4 microRNAs, consistent with reports that microRNAs are more robust to RNA degradation compared to mRNA [22], perhaps because they are more thermodynamically stable than mRNAs.

Without excluding any genes, we then compared the orthogonal approaches of either excluding quality-flagged arrays or compensating for RNA degradation at different steps in the analysis. Sample clustering showed that when using ComBat adjustment, quality-flagged samples no longer clustered together. Furthermore, samples tend to segregate more clearly by disease status following adjustment, which suggests that the algorithm is not introducing artifacts. It is worth noting that patients 13, 4 and 18 were diagnosed with a hereditary form of CRC (HNPCC) – it is therefore not surprising that the ‘normal’ samples from these patients form a separate cluster.

Irrespective of sample/array quality, applying compensatory measures for RNA degradation performed at least as well as excluding arrays that were flagged during quality assessment, as judged by gene expression analysis and IPA. At a p-value of 0.01, SVA and Combat detected the highest number of differentially expressed genes between tumour and normal samples and the top four methods applied here had 1117 differentially expressed genes in common. To evaluate the biological plausibility of the genes deemed significantly differentially expressed between tumour and normal samples, we harnessed the results from IPA to show that, in terms of the top scoring biological functions and upstream regulators, there is considerable overlap in the identity and direction of biological activation when comparing analysis methods that either excluded or included quality-flagged arrays. These results suggest that our analysis strategies are biologically sound and not biased by non-biological variance.

The relevance of each method will depend on the downstream application and the proportion of quality-flagged arrays: If a small percentage of arrays are flagged, there might not be much benefit in including them for downstream analysis. However, if a large proportion of the arrays are affected by RNA quality – which is likely to often be the case where the RNA is derived from irreplaceable historical clinical samples – the ability to retain all arrays and to account for these effects in the analysis will be valuable. Here, ComBat may be useful if direct data adjustment is required, e.g. for sample/gene clustering. On the other hand, for analysis of differential expression, especially when the source of non-biological variance is not immediately apparent, SVA may be most useful since it does not require supervision; notably, in our hands SVA was able to identify two surrogate variables which closely corresponded to “batch” and “quality” factors, judged by the grouping of samples. To establish whether the measures used here to compensate for quality-effects are superior to excluding these arrays from the analysis will require a controlled study with known true- and false-positives where the discriminatory power of each method can be objectively investigated. However, the significant overlap observed between the differentially expressed genes identified by the different approaches used here, combined with the considerable overlaps in both biological function and upstream regulators identified by pathway analysis of the resultant data, argues against a simple expansion of false positives when lower quality array data is included in the analyses. The quality assessment and data analysis methods discussed here should in principle be as useful for Affymetrix Exon ST array analysis as well.

## Conclusions

In conclusion, array quality measures can be used to set quality thresholds, to provide valuable information that can be used to improve the linear model of differential expression, or to correct expression signal prior to assessing differential expression. We suggest that accounting for known or unknown sources of variation, such as variable RNA integrity and batch, by implementing ComBat or Surrogate Variable Analysis for analysis of differential gene expression enables robust analysis of microarray datasets derived from variable and low quality RNA, thereby extending the range of clinical samples that are suitable for microarray analysis.

## Methods

### Sample collection and storage

Paired colorectal patient samples (diseased tumour tissue and adjacent healthy gut epithelial tissue) were collected during surgical resection of previously untreated patients at the Groote Schuur Hospital, Cape Town, South Africa. The samples were frozen immediately in liquid nitrogen and stored at -80°C. Ethical consent was obtained (UCT HREC REF 416/2005) and each patient provided written informed consent to donate samples from the tissues left over after surgical resection to subsequent molecular studies.

### Sample preparation and quality control

Frozen samples were transitioned to RNA®later-ICE (Ambion), an RNA stabilisation solution, using dry ice to prevent thawing of the tissue at any stage. RNA was extracted using a Dounce homogenizer and the AllPrep DNA/RNA/Protein kit (Qiagen) including DNAse treatment. RNAseZap (Ambion) was used to eliminate RNAse from the work surface, pipettes and glassware. RNA integrity assessment was conducted on an Agilent Bioanalyser 2100.

### Quantitative real-time PCR

From a biological perspective, we used the stability of expression of housekeeping genes to investigate the effect of RNA integrity on array- and qRT-PCR performance. Gene candidates were selected from those previously been specifically identified as good reference genes for colorectal cancer [17-21]. Expression stabilities were ranked using the Normfinder algorithm [23] and three genes were selected for use as reference genes. All primers except those for *b2m*[24] were designed using Primer-BLAST - sequences are shown in Table 4. Experiments were performed in triplicate on a Roche LightCycler® 480 Real-Time PCR System in 96-well format. Efficiency was determined for each primer pair using a two-fold dilution series across five points for five patient samples of varying RNA integrity. For each patient, tumour vs. normal fold change was determined based on the method of Antonov et al whereby the Ct of the test gene is normalised by the geometric mean of multiple control genes [9]. Since our efficiencies were quite low in some cases, we adapted the Antonov et al method to include primer efficiency as shown in the equation below:

$$\frac{e^{\Delta \mathrm{Ct}\left(t\right)}}{\sqrt[{\;e_{i}^{\Delta \mathrm{Ct}\left(i\right)}\times e_{i+1}^{\Delta \mathrm{Ct}\left(i+1\right)}\ldots \times e_{i+n}^{\Delta \mathrm{Ct}\left(i+n\right)}}]{n+1}}$$

where *t* represents the test gene, *e* represents efficiency and *i* represents the control gene(s).

**Table 4:** Primers used for qRT-PCR analysis

**Test genes**	**Forward primers (5' - 3')**	**Reverse primers (5' - 3')**	**Product (bp)**
dpep1	GACAACTGGCTGGTGGACA	ACCACACGCTGCCCAAA	74
cldn1	GCTGTCATTGGGGGTGCGAT	GGCAACTAAAATAGCCAGACCTGC	54
**Reference genes**			
ubc	GGTCGCAGTTCTTGTTTGTGG	CACGAAGATCTGCATTGTCAAG	59
b2m	TGCTGTCTCCATGTTTGATGTATCT	TCTCTGCTCCCCACCTCTAAGT	86
atp5e	CTGGACTCAGCTACATCCGA	GCATCTCTCACTGCTTTTGCAC	55

### Microarray analysis: Affymetrix HuGene 1.0 ST expression arrays

Thirty-four samples with A260/230 ratios of at least 1.6, RINs of at least 2 and no sign of genomic DNA contamination, were selected for microarray analysis. The samples were amplified from 200ng of total RNA in accordance with the Ambion® WT Expression assay kit and fragmented and end labeled in accordance with the Affymetrix® GeneChip® WT Terminal Labeling protocol. The prepared targets were hybridized overnight to Affymetrix Human Gene 1.0 ST arrays. Following hybridization, the arrays were washed and stained using the GeneChip Fluidics Station 450 and scanned using the GeneChip® Scanner 3000 7G. Arrays were processed in two batches - batch one had 10 arrays, and batch two 24. Individual patient pairs were not split across batches.

### Microarray quality assessment and data analysis

Standard Affymetrix quality control was conducted using Expression Console® Software: The quality of cDNA preparation and array hybridisation was assessed using appropriate spike-in controls at each stage.

Raw array quality was investigated at the probe level by 1) the difference between the mean of the perfect match probes and the mean of the background probes for each array as well as 2) the coefficient of variation (CV) across all probes for each array. A threshold for the CV across probes was set as two standard deviations from the mean CV, where the mean was calculated from arrays with RINs > 6. The data was preprocessed in R using the Bioconductor packages frma [25], oligo [26], and the ComBat algorithm for batch correction [12]. Preprocessed data quality was assessed using the global normalised, unscaled standard error (GNUSE) [14]. The SE estimates are normalized such that for each probe set, the median standard error across all arrays is equal to 1. Since most genes are not expected to be differentially expressed, boxplots for each array should be centered around 1. Samples with a median GNUSE of greater than 1.25 were flagged for downstream analysis. This threshold is fairly arbitrary and has not been validated for the Gene 1.0 ST platform but roughly equates to having a precision that is on average 25% worse than the average Gene 1.0 ST array [14].

Five comparative methods for analysis of differential expression were individually applied to the preprocessed data: 1) The arrayWeights function in the Bioconductor package limma [27] was used to estimate array quality weights which were then included in the linear model fit; 2) Arrays that were flagged in array quality assessment were excluded from the analysis; 3) The ComBat algorithm [12] for batch correction was applied to directly adjust the data according to quality, where arrays were divided into two categories according to the array quality assessment; 4) “Quality” and “batch” were included as a factors in the linear model together with disease status; 5) Surrogate variable analysis was applied to frma-processed data without any direct adjustment, the output from SVA being incorporated into the linear model fit [13].

To rank genes by evidence for differential expression, the eBayes function in limma was applied to compute moderated t-statistics, moderated F-statistic, and log-odds of differential expression by empirical Bayes shrinkage of the standard errors towards a common value [27]. Next, using the topTable function in limma, p-values were adjusted for multiple hypothesis testing using the Benjamini and Hochberg method [28]. Transcript clusters were annotated in R using the Bioconductor package hugene10sttranscriptcluster.db (Affymetrix Human Gene 1.0-ST Array Transcriptcluster Revision 8 annotation data, assembled using data from public repositories).

The subset of genes differentially affected by RNA quality was similarly obtained, now using array quality for grouping, instead of disease status. Genes with adjusted p-values ≤ 0.05 and FCs ≥ |2| were included in the analysis. Transcript length was obtained for all annotated transcript clusters using the Bioconductor package goseq [29]. Hierarchical clustering with average linkage and Euclidian distance as distance measure was performed in R using the hclust function.

For Ingenuity Pathway Analysis, genes that were found to be significantly differentially expressed for each method (adjusted p-value ≤ 0.01), were used as input for IPAs “Core Analysis”. Here, statistically significant over-representation of our listed genes in a given process such as “colorectal tumour” or “infection of embryonic cell lines” is scored by p-value, using the right-tailed Fisher’s Exact Test. In the case of upstream regulators, the predicted activation state and activation z-score is based on the direction of fold change values for genes in the input dataset for which an experimentally observed causal relationship has been established. Performance was assessed using the top 10 functions in terms of p-values for each method while taking into account the relevance of the function to colorectal cancer.

## Competing interests

The authors declare that they have no competing interests.

## Authors’ contributions

KV carried out the sample preparation and RNA extraction and performed the data analysis. KV and JB conceived and designed the study and wrote the manuscript. Both authors read and approved the final manuscript.

## References

[B1] Tomita H Vawter MP Walsh DM Evans SJ Choudary PV Li J Overman KM Atz ME Myers RM Jones EG Watson SJ Akil H Bunney WE Effect of agonal and postmortem factors on gene expression profile: quality control in microarray analyses of postmortem human brain Biological Psychiatry 2004 55 4 346 352 10.1016/j.biopsych.2003.10.013 14960286 PMC3098566

[B2] Mengual L Burset M Ars E Ribal MJ Lozano JJ Minana B Sumoy L Alcaraz A Partially Degraded RNA from Bladder Washing is a Suitable Sample for Studying Gene Expression Profiles in Bladder Cancer European Urology 2006 50 1347 1356 10.1016/j.eururo.2006.05.039 16815626

[B3] Linton KM Hey Y Saunders E Jeziorska M Denton J Wilson CL Swindell R Dibben S Miller CJ Pepper SD Radford JA Freemont AJ Acquisition of biologically relevant gene expression data by Affymetrix microarray analysis of archival formalin-fixed paraffin-embedded tumours British Journal of Cancer 2008 98 1403 1414 10.1038/sj.bjc.6604316 18382428 PMC2361698

[B4] Linton K Hey Y Dibben S Miller C Freemont A Radford J Pepper S Methods comparison for high-resolution transcriptional analysis of archival material on Affymetrix Plus 2.0 and Exon 1.0 microarrays BioTechniques 2009 47 587 596 10.2144/000113169 19594443

[B5] April C Klotzle B Royce T Wickham-garcia E Boyaniwsky T Izzo J Cox D Jones W Rubio R Holton K Matulonis U Quackenbush J Fan J Whole-Genome Gene Expression Profiling of Formalin-Fixed, Paraffin-Embedded Tissue Samples PloS one 2009 4 12 1 10 10.1371/journal.pone.0008162 PMC2780295 19997620

[B6] Opitz L Salinas-riester G Grade M Jung K Jo P Emons G Ghadimi BM Beiÿ barth T Gaedcke J Impact of RNA degradation on gene expression profiling BMC Medical Genomics 2010 3 36 1 14 20696062 10.1186/1755-8794-3-36 PMC2927474

[B7] Fleige S Pfaffl MW RNA integrity and the effect on the real-time qRT-PCR performance Molecular aspects of medicine 2006 27 126 139 10.1016/j.mam.2005.12.003 16469371

[B8] Lassmann S Kreutz C Schoepflin A Hopt U Timmer J Werner M A novel approach for reliable microarray analysis of microdissected tumor cells from formalin-fixed and paraffin-embedded colorectal cancer resection specimens Journal of molecular medicine 2009 87 211 224 10.1007/s00109-008-0419-y 19066834

[B9] Antonov J Goldstein DR Oberli A Baltzer A Pirotta M Fleischmann A Altermatt HJ Jaggi R Reliable gene expression measurements from degraded RNA by quantitative real-time PCR depend on short amplicons and a proper normalization Laboratory Investigation 2005 85 1040 1050 10.1038/labinvest.3700303 15951835

[B10] Binder H Krohn K Preibisch S "Hook"-calibration of GeneChip-microarrays: chip characteristics and expression measures Algorithms for molecular biology 2008 **3:**11. 10.1186/1748-7188-3-11 PMC2543012 18759984

[B11] Chow ML Winn ME Li HR April C Wynshaw-Boris A Fan JB Fu X Courchesne E Schork NJ Preprocessing and quality control strategies for Illumina DASL assay-based brain gene expression studies with semi- degraded samples Frontiers in Genetics 2008 3 11 22375143 10.3389/fgene.2012.00011 PMC3286152

[B12] Johnson WE Li C Adjusting batch effects in microarray expression data using empirical Bayes methods Biostatistics 2007 8 118 127 10.1093/biostatistics/kxj037 16632515

[B13] Leek JT Storey JD Capturing Heterogeneity in Gene Expression Studies by Surrogate Variable Analysis PLoS Genetics 2007 3 9 1724 1735 17907809 10.1371/journal.pgen.0030161 PMC1994707

[B14] McCall MN Murakami PN Lukk M Huber W Irizarry R Assessing affymetrix GeneChip microarray quality BMC bioinformatics 2011 **12:**137. 10.1186/1471-2105-12-137 PMC3097162 21548974

[B15] Gibbons FD Roth FP Judging the Quality of Gene Expression-Based Clustering Methods Using Gene Annotation Genome Research 2002 12 1574 1581 10.1101/gr.397002 12368250 PMC187526

[B16] Dalman MR Anthony D Gayathri N Zhong-Hui D Fold change and p-value cutoffs significantly alter microarray interpretations BMC Bioinformatics 2012 13 Suppl 2 S11 10.1186/1471-2105-13-S2-S11 22536862 PMC3305783

[B17] Salazar R Roepman P Capella G Moreno V Simon I Dreezen C Lopez-Doriga A Santos C Marijnen C Westerga J Bruin S Kerr D Kuppen P van de Velde C Morreau H Van Velthuysen L Glas AM Van't Veer LJ Tollenaar R Gene expression signature to improve prognosis prediction of stage II and III colorectal cancer Journal of clinical oncology 2011 29 17 24 10.1200/JCO.2010.30.1077 21098318

[B18] O'Connell MJ Lavery I Yothers G Paik S Clark-Langone KM Lopatin M Watson D Baehner FL Shak S Baker J Cowens JW Wolmark N Relationship between tumor gene expression and recurrence in four independent studies of patients with stage II/III colon cancer treated with surgery alone or surgery plus adjuvant fluorouracil plus leucovorin Journal of clinical oncology 2010 28 25 3937 3944 10.1200/JCO.2010.28.9538 20679606 PMC2940392

[B19] Dydensborg AB Herring E Auclair J Tremblay E Beaulieu JF Normalizing genes for quantitative RT-PCR in differentiating human intestinal epithelial cells and adenocarcinomas of the colon American Journal of Gastrointestinal and Liver Physiology 2006 290 G1067 G1074 10.1152/ajpgi.00234.2005 16399877

[B20] Rubie C Kempf K Hans J Su T Tilton B Georg T Brittner B Ludwig B Schilling M Housekeeping gene variability in normal and cancerous colorectal, pancreatic, esophageal, gastric and hepatic tissues Molecular and cellular probes 2005 19 101 109 15680211 10.1016/j.mcp.2004.10.001

[B21] Kheirelseid EH Chang KH Newell J Kerin MJ Miller N Identification of endogenous control genes for normalisation of real-time quantitative PCR data in colorectal cancer BMC molecular biology 2010 **11:**12. 10.1186/1471-2199-11-12 PMC2825202 20122155

[B22] Jung M Schaefer A Steiner I Kempkensteffen C Stephan C Erbersdobler A Jung K Robust microRNA stability in degraded RNA preparations from human tissue and cell samples Clinical chemistry 2010 56 6 998 1006 10.1373/clinchem.2009.141580 20378769

[B23] Andersen CL Jensen JL Ørntoft TF Normalization of real-time quantitative reverse transcription-PCR data: a model-based variance estimation approach to identify genes suited for normalization, applied to bladder and colon cancer data sets Cancer research 2004 64 15 5245 5250 10.1158/0008-5472.CAN-04-0496 15289330

[B24] Vandesompele J Preter KD Poppe B Roy NV Paepe AD Accurate normalization of real-time quantitative RT -PCR data by geometric averaging of multiple internal control genes Genome biology 2002 3 7 1 12 10.1186/gb-2002-3-7-research0034 PMC126239 12184808

[B25] Mccall MN Bolstad BM Irizarry RA Frozen robust multiarray analysis (fRMA) Biostatistics 2010 11 2 242 253 10.1093/biostatistics/kxp059 20097884 PMC2830579

[B26] Carvalho B Irizarry RA Scharpf RB Carey VJ Processing and Analyzing Affymetrix SNP Chips with Bioconductor Stat Biosci 2009 1 160 180 10.1007/s12561-009-9015-0

[B27] Smyth GK Linear Models and Empirical Bayes Methods for Assessing Differential Expression in Microarray Experiments Statistical Applications in Genetics and Molecular Biology 2004 **3:**3. 10.2202/1544-6115.1027 16646809

[B28] Benjamini Y Hochberg Y Controlling the False Discovery Rate: a Practical and Powerful Approach to Multiple Testing Journal of the Royal Statistical Society 1995 57 289 300

[B29] Young MD Wakefield MJ Smyth GK Oshlack A Gene ontology analysis for RNA-seq: accounting for selection bias Genome Biology 2010 11 R14 10.1186/gb-2010-11-2-r14 20132535 PMC2872874

